# Three-Dimensional Printed Silk Fibroin/Hyaluronic Acid Scaffold with Functionalized Modification Results in Excellent Mechanical Strength and Efficient Endogenous Cell Recruitment for Articular Cartilage Regeneration

**DOI:** 10.3390/ijms251910523

**Published:** 2024-09-29

**Authors:** Weili Shi, Jiahao Zhang, Zeyuan Gao, Fengyi Hu, Simin Kong, Xiaoqing Hu, Fengyuan Zhao, Yingfang Ao, Zhenxing Shao

**Affiliations:** 1 Department of Sports Medicine, Peking University Third Hospital, Institute of Sports Medicine of Peking University, Beijing 100191, China; shiweilixmu@bjmu.edu.cn (W.S.); jiahaozhang_pkmu@163.com (J.Z.); gzy9507@hsc.pku.edu.cn (Z.G.); hfy1322@pku.edu.cn (F.H.); 2211210388@stu.pku.edu.cn (S.K.); huxiaoqingbd01@sina.com (X.H.); mickeyzhaofy@163.com (F.Z.); 2 Beijing Key Laboratory of Sports Injuries, Beijing 100191, China; 3 Engineering Research Center of Sports Trauma Treatment Technology and Devices, Ministry of Education, Beijing 100191, China

**Keywords:** three-dimensional printing, double-network scaffolds, cell recruitment, bone marrow mesenchymal stem cell, cartilage repair

## Abstract

Treatment of articular cartilage remains a great challenge due to its limited self-repair capability. In tissue engineering, a scaffold with both mechanical strength and regenerative capacity has been highly desired. This study developed a double-network scaffold based on natural biomaterials of silk fibroin (SF) and methacrylated hyaluronic acid (MAHA) using three-dimensional (3D) printing technology. Structural and mechanical characteristics of the scaffold was first investigated. To enhance its ability of recruiting endogenous bone marrow mesenchymal stem cells (BMSCs), the scaffold was conjugated with a proven BMSC-specific-affinity peptide E7, and its biocompatibility and capacity of cell recruitment were assessed in vitro. Animal experiments were conducted to evaluate cartilage regeneration after transplantation of the described scaffolds. The SF/HA scaffolds exhibited a hierarchical macro-microporous structure with ideal mechanical properties, and offered a 3D spatial microenvironment for cell migration and proliferation. In vitro experiments demonstrated excellent biocompatibility of the scaffolds to support BMSCs proliferation, differentiation, and extracellular matrix production. In vivo, superior capacity of cartilage regeneration was displayed by the SF/MAHA + E7 scaffold as compared with microfracture and unconjugated SF/MAHA scaffold based on macroscopic, histologic and imaging evaluation. In conclusion, this structurally and functionally optimized SF/MAHA + E7 scaffold may provide a promising approach to repair articular cartilage lesions in situ.

## 1. Introduction

Cartilage defects are a common and challenging problem due to its limited self-repair capacity [1,2]. Consequently, chondral lesions often lead to osteoarthritis and the progressive degeneration of the joint [1]. Currently, the mainstay treatments for cartilage injuries include arthroscopic debridement, bone marrow stimulation, autologous chondrocyte transplantation, and matrix-induced autologous chondrocyte transplantation [2]. However, these methods mentioned above still have some limitations, such as the generation of fibrocartilage, the need for a second surgery, and high costs [3].

Tissue engineering has shown great potential in cartilage repair. By providing a well-differentiated microenvironment while maintaining mechanical properties, tissue-engineered scaffolds can enhance the chondrogenic differentiation of bone marrow mesenchymal stem cells (BMSCs). These scaffolds consist of highly biocompatible natural biomaterials like hyaluronic acid and chitosan, as well as synthetic materials such as polycaprolactone and polylactic acid. Despite controllable and reproducible production, synthetic polymeric materials raise concerns over foreign body reaction and toxicity of degradation by-products. In recent years, there has been a growing focus on natural biomacromolecules due to their inherent advantages in biochemical and biophysical properties. These advantages include renewability, nontoxicity, biocompatibility and biodegradability [4].

In addition, the structure of biological scaffold is also very important for tissue engineering cartilage repair. Three-dimensional (3D) printing is considered a crucial technological advancement in material engineering [5]. Three-dimensional printing, also referred to as additive manufacturing, has revolutionized the world of manufacturing and design over the past few decades [6]. The 3D spatial scaffold has been reported to provide a supportive microenvironment for BMSC attachment, proliferation and differentiation [7]. A porous structure with appropriate pore size helps to optimize the efficacy of the functional scaffold incorporating endogenous BMSCs [8]. The 3D scaffold used in regenerative medicine should have the following characteristics: biocompatibility, which means that they should be compatible with the human body; biodegradability; and appropriate mechanical properties [9]. The materials currently used in the field of 3D printing struggle to combine the above features [10,11].

Silk fibrin (SF) is a natural biopolymer with outstanding mechanical properties, excellent biocompatibility and process-ability. In recent years, SF has been demonstrated with great potential for cartilage tissue engineering [10,11]. Previous studies have shown that SF hydrogel can be constructed by self-assembly of β-sheets [12], and that SF contains many amine groups, which is conducive to develop a functional scaffold. Hyaluronic acid (HA) is a non-sulfated glycosaminoglycan composed of repeating disaccharide units. These units consist of β-d-glucuronic acid and N-acetyl-d-glucosamine, linked alternately by β-1,3 and β-1,4 glycosidic bonds [13]. As an important component of the extracellular matrix (ECM) of cartilage, HA possesses advantages of being highly printable and biocompatible. However, its application is hindered by its deficiency of fast degradation and inadequate strength. In this study, we modified HA with methacrylic anhydride in an alkali environment to synthesize methacrylated hyaluronic acid (MAHA), which contributes to maintaining a rigid network. The combined use of SF and MAHA not only provides biocompatible composite hydrogels suitable for 3D printing [14], but also increases the mechanical properties for scaffold establishment.

Recruiting and retaining BMSCs in the injured area remains an unsettled issue for cartilage repair. A number of studies have explored the addition of biologic factors to scaffolds to impart the capacity of cell recruitment [8,14]. In a previous work by Shao et al. [15], a seven-amino-acids peptide sequence (E7) was identified using phage display technology. This sequence showed high specific affinity to bone marrow-derived MSCs. In addition, the E7 peptitide could exert pro-chondrogenic effects on BMSCs. Therefore, in this study, we conjugated E7 as the key biologic factor onto the SF/MAHA scaffold to promote in situ BMSCs recruitment.

**Figure 1:**
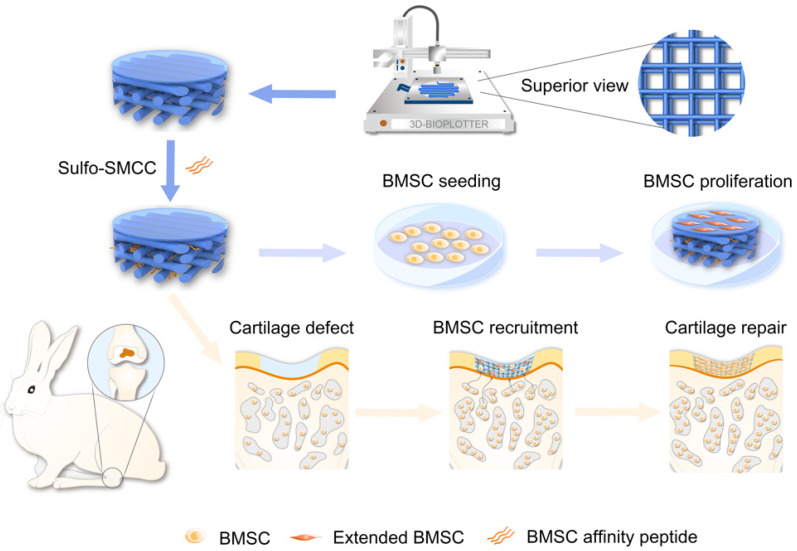
Schematic illustration of the study design. In this study, the scaffold was manufactured by three-dimensional printing technology, with E7 peptide conjugated to the scaffold to impart the capacity of stem cells recruitment. The scaffold was co-cultured with BMSCs in vitro to evaluate its biocompatibility, and then it was implanted in vivo to assess BMSCs recruitment for cartilage repair.

In this study, we developed an E7-modified SF/MAHA composite scaffold with excellent mechanical properties and efficient cell-recruiting ability to repair chondral lesions in situ (Figure 1). The porous and 3D spatial structure of this SF/MAHA + E7 scaffold provides suitable microenvironment for BMSCs, promoting their proliferation, differentiation and ECM production during hyaline cartilage regeneration. Experiments were conducted to investigate the scaffold’s potential in synergistically promoting chondrogenesis in vitro, stimulating cartilage regeneration, and facilitating joint function recovery in vivo.

## 2. Results

### 2.1. Description of the Main Results

#### 2.1.1. Preparation of SF/MAHA Ink for 3D Printing

**Figure 2:**
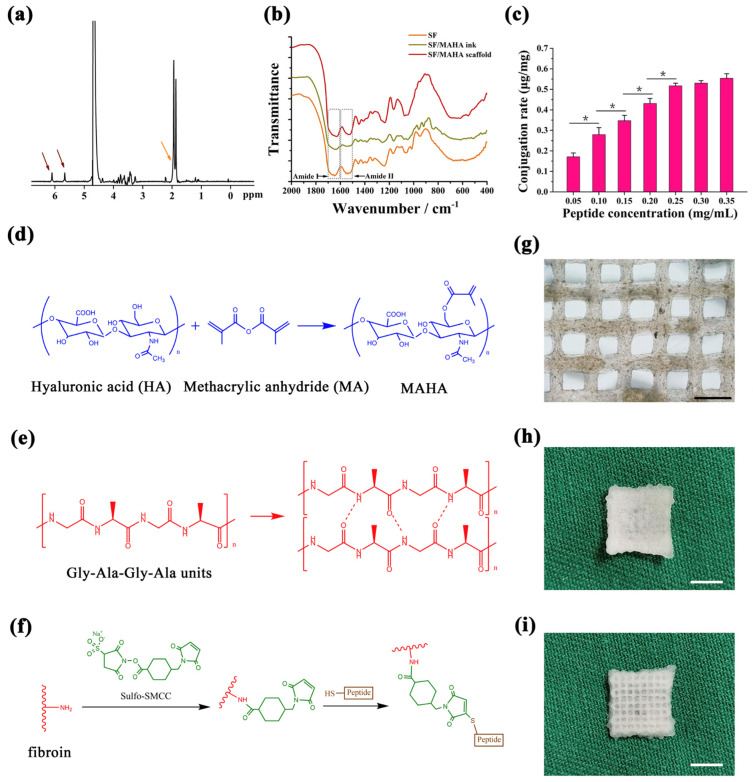
Crosslinking procedures and gross structure of the scaffolds. (**a**) NMR profile of MAHA. (**b**) FTIR spectra of SF hydrogel, SF/MAHA ink, and SF/MAHA scaffolds. (**c**) Quantification of peptide conjugated to SF/MAHA scaffolds. (* *p* < 0.05) (**d**) MAHA synthesis by modifying HA with methacrylic anhydride under an alkaline environment. (**e**) Secondary structural changes of SF after ethanol treatment. (**f**) Conjugation of E7 peptide onto the scaffolds. (**g**) Inverted microscope view of the scaffolds (scale bar = 1 mm). (**h**) Gross appearance of the micro-porous membrane on top of the scaffolds (scale bar = 5 mm). (**i**) Gross appearance of the porous structure of the scaffolds (scale bar = 5 mm). FTIR, Fourier transform infrared spectroscopy. MAHA, methacrylated hyaluronic acid. SF, silk fibroin.

In order to print SF/MAHA scaffold, we first synthesized a double-network hydrogel as our extrusion-printing ink based on Xiao’s work [16]. HA was modified with methacrylic anhydride in an alkali environment to synthesize MAHA (the methacrylated degree of HA was about 13%) [17], which functioned as the precursor of the soft network (Figure 2a,d), and offered resistance against crack propagation. SF hydrogel was chosen as the rigid network via photo-initiated free radical polymerization. After UV treatment, main peaks of fibroin in amide I and II transformed from 1650 cm^−1^ and 1534 cm^−1^ to 1627 cm^−1^ and 1517 cm^−1^, demonstrating a structural transition of fibroin chains from amorphous state to β-sheet secondary structure (Figure 2b). While being photo-crosslinked under UV light, MAHA and fibroin were simultaneously polymerized and formed a transparent hydrogel, which was used as the bioink for extrusion-based printing. After exploring the experimental conditions suitable for fluent 3D printing, 0.35% MAHA and 1% to 5% SF (both were dissolved in DI water) were chosen for the following work.

#### 2.1.2. Chemical Conjugation of BMSC Affinity Peptide

To enhance the mechanical properties of 3D printed hydrogel, SF/MAHA hydrogel was lyophilized and immersed in ethanol, inducing the self-assembly of SF into silk II crystalline structure (Figure 2e). A proven BMSC affinity peptide EPLQLKM-C (E7) was conjugated on SF/MAHA scaffolds for BMSC recruitment by an amine-sulfhydryl crosslinker Sulfo-SMCC (Figure 2f) [18,19]. The saturated concentration of peptide modification was detected through measuring the fluorescence intensity of the FITC-labeled E7 crosslinked on scaffolds. The result showed that the modification degree of FITC-E7 reached its maximum when the scaffold was immersed in a 0.25 mg/mL peptide solution (Figure 2c).

#### 2.1.3. Design and Preparation of 3D Spatial Structure of the Scaffolds

**Figure 3:**
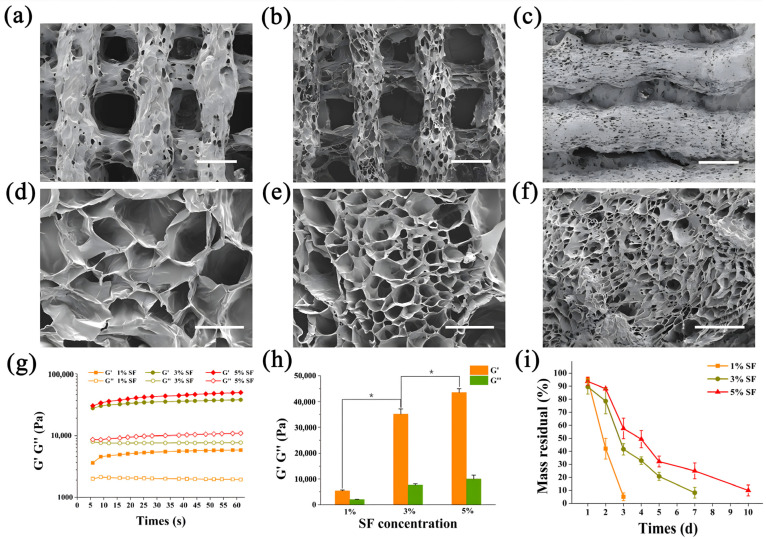
Three-dimensional spatial structure and characterization of the scaffolds. (**a**–**c**) SEM images of scaffolds formed at −20 °C, −80 °C and −178 °C (scale bar = 500 μm). (**d**–**f**) Enlarged view of (**a**–**c**) (scale bar = 100 μm). (**g**,**h**) Storage modulus (G′) and loss modulus (G″) of SF/MAHA scaffolds under different SF concentrations (* *p* < 0.05); (**i**) Enzymatic degradation of SF/MAHA scaffolds under different SF concentrations. MAHA, methacrylated hyaluronic acid. SEM, scanning electron microscopy. SF, silk fibroin.

To better simulate the spatial structure of articular cartilage, 3D printing and phase separation technology were incorporated to create a hierarchical porous structure (Figure 2i). General and microscopic observations of the grid construct showed that the bioink formed even squares between the SF/MAHA strands (Figure 2g). The size of macro-pores was determined by the digital model of 3D printed scaffolds, while the size of micro-pores was determined by the annealing temperature of lyophilization. A membrane with only micro-pores was designed on the top of the scaffolds to simulate the superficial layer of cartilage, which also helped to prevent BMSC from diffusing out of the scaffolds (Figure 2h). As previously reported, cell proliferation in scaffolds is enhanced with a pore size around 400 μm [20,21]. Therefore, we designed the SF/MAHA scaffold with a uniform pore size of 400 μm and produce the optimized structure via 3D printing. When scaffolds were separately annealed at different temperatures, results showed that a strict obturator structure was formed at −20 °C (Figure 3a,d). The inner connectivity of pores was improved when the annealing temperature was set at −80 °C (Figure 3b,e) and −178 °C (Figure 3c,f). Since the distribution of pore size was centered below 10 μm at −178 °C, which was too small for cell migration compared with the size of cells, we chose −80 °C as the cryo-storage temperature.

#### 2.1.4. Mechanical Properties and Degradation of SF/MAHA Scaffolds

To screen ideal SF concentration for cartilage regeneration, mechanical behaviors and enzymatic degradation of SF/MAHA scaffolds were assessed. The storage modulus was constantly higher than the loss modulus according to the oscillatory time sweep test, indicating good elastic property and stability of the scaffolds. Both of the storage modulus and the loss modulus of the scaffolds rose with the increase of SF concentration, while the improvements became minimal between 3% SF group and 5% SF group (Figure 3g,h). The increase of SF concentration also extended the degradation time of the scaffolds (Figure 3i). Based on the results above, SF concentration was set at 3% as the printing parameter for the scaffolds, which storage modulus is higher than that of conventional biomedical scaffolds [18].

#### 2.1.5. Capacity of Cell Recruitment and Cell Viability In Vitro

**Figure 4:**
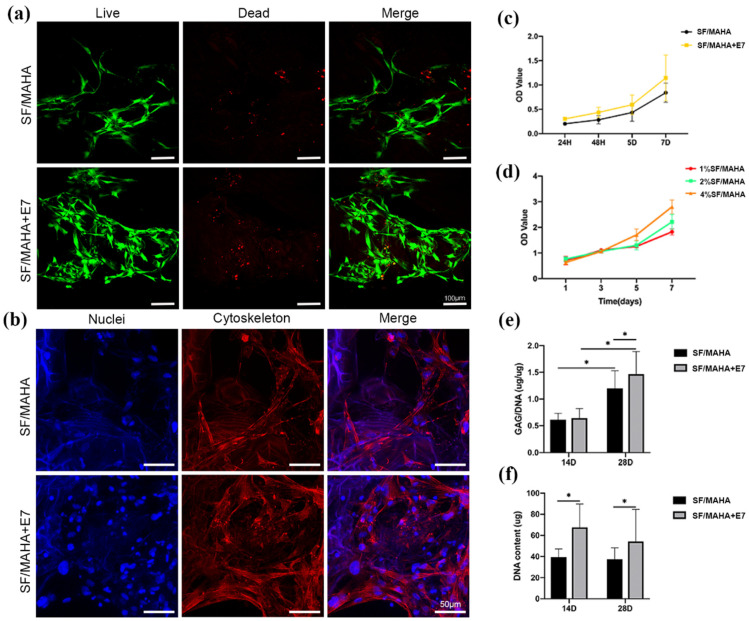
In vitro BMSCs recruitment, proliferation and matrix production of the scaffolds. (**a**) Viability of BMSCs was analyzed by Live/Dead assay (live cells: green, dead cells: red) (scale bar = 100 μm). (**b**) Morphology of BMSCs was observed via Phalloidin/Hoechst assay under confocal microscopy (cytoskeleton: red, nuclei: blue) (scale bar = 50 μm). (**c**,**d**) The viability of BMSCs seeded on different scaffolds were evaluated with CCK-8 assay. (**e**) The GAG/DNA content in different scaffolds was measured after 14 and 28 days of chondrogenic incubation. (**f**) The DNA content in different scaffolds was quantified via Hoechst 33258 assay after 14 and 28 days of incubation (* *p* < 0.05). BMSCs, bone marrow stem cells. CCK-8, cell counting kit-8. MAHA, methacrylated hyaluronic acid. SF, silk fibroin.

To evaluate the bio-function of the described scaffolds, viability and of BMSCs was analyzed using CCK-8 assay, Live/Dead assay and Phalloidin/Hoechst assay. CCK-8 assay demonstrated favorable proliferative activity of BMSCs co-cultured with both SF/MAHA and SF/MAHA + E7 scaffolds (Figure 4c,d). After seeding on the scaffolds for 3 days, BMSCs were observed under confocal microscopy. It was further confirmed that cells grew well on both scaffolds with typical fusiform morphology, and superior results of cell recruitment were observed in the SF/MAHA + E7 group (Figure 4a,b). Further, after chondrogenic incubation for 14 and 28 days, GAG production was measured and normalized by DNA content to quantify cartilaginous matrix production. GAG deposition in both groups at day 28 was significantly higher than that at day 14. Moreover, the SF/MAHA + E7 group led to significantly higher deposition of GAG than the SF/MAHA group at day 28 (Figure 4e). Also, the SF/MAHA + E7 group had more DNA content than the SF/MAHA group at both day 14 and day 28 (Figure 4f). Overall, these results demonstrated that the SF/MAHA + E7 scaffold is an ideal platform for the growth, proliferation, and ECM secretion of BMSCs.

#### 2.1.6. Evaluation of Cartilage Regeneration In Vivo

**Figure 5:**
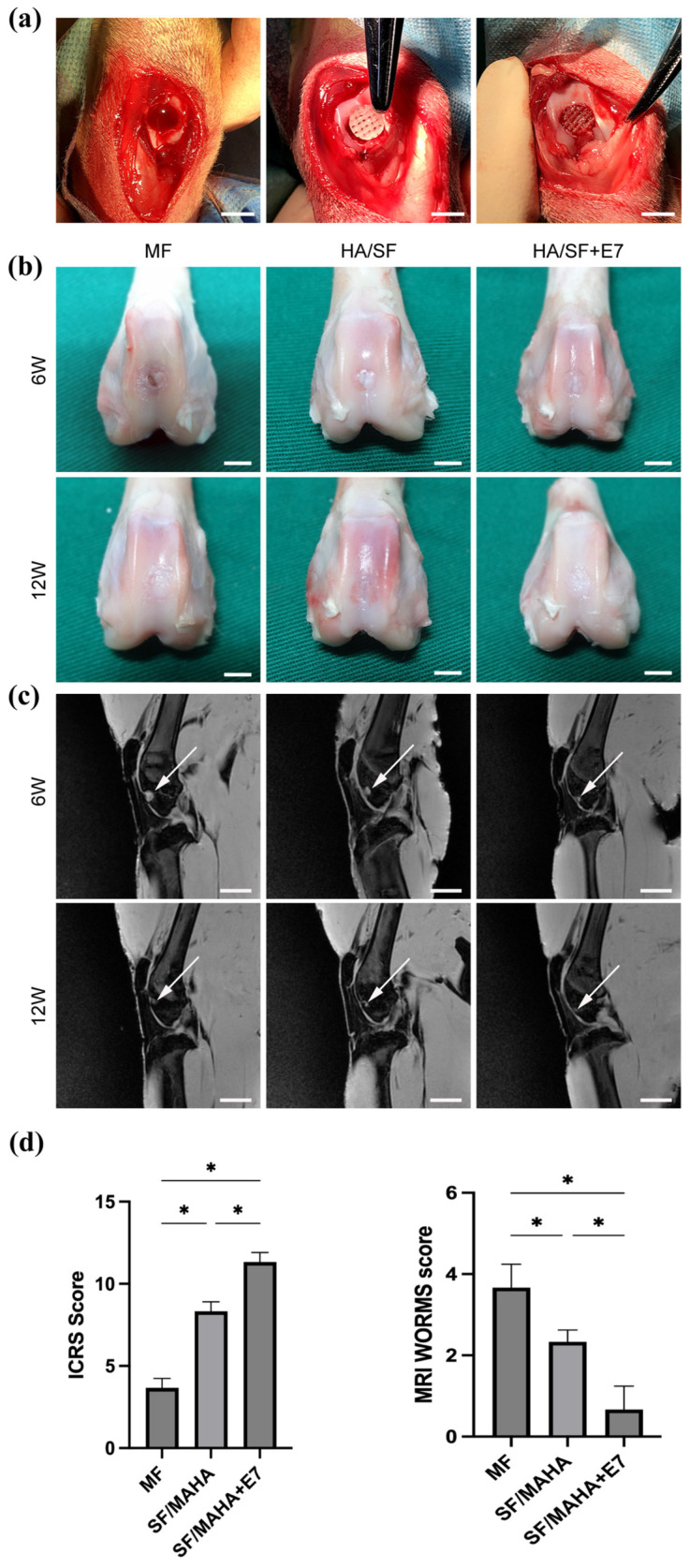
(**a**) in vivo experimental surgery to repair the cartilage defect (scale bar = 1 cm). (**b**) Macroscopic observation of the repaired area at 6 and 12 weeks after surgery (scale bar = 1 cm). (**c**) Magnetic resonance imaging examination of specimens at 6 and 12 weeks after surgery(white arrow, repaired sites) (scale bar = 2 cm). MAHA, methacrylated hyaluronic acid. SF, silk fibroin. (**d**) ICRS macroscopic score evaluating the quality of the regenerated chondral tissue (*n* = 3); WORMS score evaluating the quality of the repaired chondral tissue (*n* = 3) (* *p* < 0.05).

For in vivo experiments, SF/MAHA and SF/MAHA + E7 scaffolds were used to repair cartilage injury (Figure 5a). The MF group served as the control group, which is currently the most common treatment for cartilage injury [19]. At 6 and 12 weeks post-operation, specimens were examined using MRI. The signal intensities of the injured area in the MF group were constantly lower compared to normal cartilage at both week 6 and 12, indicating inferior neocartilage formation. In the SF/MAHA and SF/MAHA + E7 groups, the signal intensities were lower than normal cartilage at week 6, but better than the control group. Macroscopic observation of the regenerated cartilage is displayed in Figure 5b. The regenerated tissue in the MF group at week 6 and 12 was white and coarse fibrous tissue, which echoed the findings based on MRI. At week 12, the defects were partially filled with neocartilage in all groups. Compared with the MF and the SF/MAHA group, the regenerated tissue in the SF/MAHA + E7 group showed flatter surfaces and lower signal intensities on MRI (Figure 5b,c). These results indicated that SF/MAHA + E7 scaffold had great capacity of cartilage regeneration in vivo.

**Figure 6:**
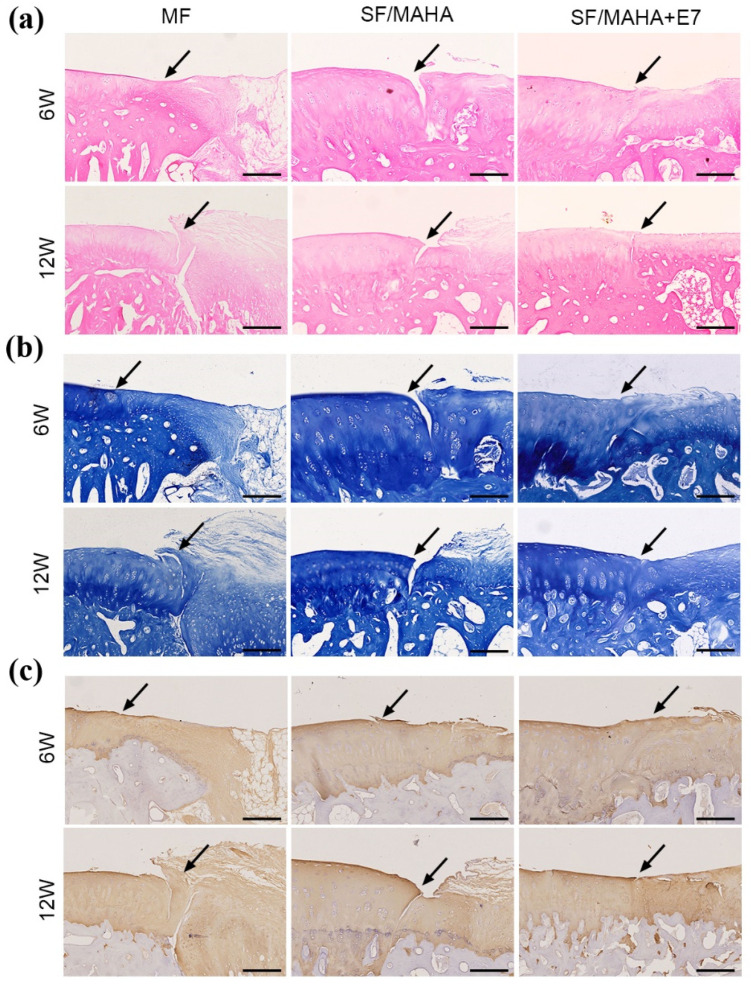
Histological assessments of neocartilage formation in the injured area. (**a**) Hematoxylin–eosin staining. (**b**) Toluidine blue staining. (**c**) Immunohistochemical staining for Col II. (the arrows indicate the margins of the normal cartilage and repaired cartilage) (Scale bar = 200 μm) MAHA, methacrylated hyaluronic acid. SF, silk fibroin.

The histological features of regenerated tissue in all groups showed no obvious inflammatory cell infiltration (Figure 6a). At 6 and 12 weeks postoperatively, the cartilage lesions remained apparent in the MF and SF/MAHA groups. At week 6, in the SF/MAHA + E7 group, the defects was filled, but the boundary between normal cartilage and regenerated tissue was visible. However, the regenerated tissue showed a flat surface and hyaline cartilage formation was observed in the HA/SF + E7 group, although a small gap between normal cartilage and regenerated tissue was observed.

In addition, toluidine blue staining of GAGs (Figure 6b) and immunohistochemical staining of COL II (Figure 6c) were performed to evaluate the quality of cartilage repair. At 6 and 12 weeks after the operation, the defects in the MF group were mostly covered with fibrous tissue. The SF/MAHA and SF/MAHA + E7 groups exhibited positive staining, but the regenerated tissue in the SF/MAHA group showed incomplete integration with the adjacent normal cartilage at both time points. In contrast, the SF/MAHA + E7 group displayed positive staining of regenerated tissue with a nearly invisible boundary between the normal and regenerated cartilage.

## 3. Discussion

In the present study, we developed a E7 peptide-modified SF/MAHA scaffold as a functionalized tissue engineering network to repair cartilage injury in situ. The main findings are as follows: (a) the double-network SF/MAHA scaffold simultaneously maintained biocompatibility and mechanical properties; (b) a hierarchical porous structure was controllably constructed via 3D printing as the basis of cell recruitment; (c) the E7-conjugated scaffold resulted in superior capacity of BMSCs recruitment, proliferation and matrix production in vitro; (d) the E7-conjugated showed better results in cartilage regeneration in vivo.

Currently, tissue engineering has become a promising treatment for cartilage defects in situ [8,20]. Hydrogels composed of natural polymers are promising biomaterials for tissue engineering due to their intrinsic cytocompatibility characteristics. However, a common challenge is the use of hydrogels based on natural polymers is their limited mechanical performance, which restricts their application in mechanically demanding fields such as cartilage, tendon, and ligament repair [20]. To overcome this challenge and meet the growing demand for biocompatible hydrogels with improved mechanical properties that mimic the ECM, we developed a dual-crosslinked SF/MAHA hydrogel scaffold. Both of the SF and HA are naturally derived biomaterials, which optimized the biocompatibility of the scaffolds. Here, SF was incorporated as a rigid network, while HA served as a soft network. Further, we modified HA with methacrylic anhydride in an alkali environment to synthesize MAHA with a methacrylated degree of 13%, which offered resistance against crack propagation and improved the mechanical strength of the scaffolds. In property assessments of the SF/MAHA scaffolds, we investigated the effect of SF concentration (1%, 3% and 5%) on mechanical behaviors and enzymatic degradation. With the increase of SF concentration, both of the storage modulus and loss modulus were improved, and the degradation time was extended of the scaffolds. This echoed the primary purpose of incorporating SF into the double-network scaffolds.

In the current study, cartilage scaffolds with a hierarchical macro-microporous structure were prepared using 3D printing technology, demonstrating highly controllable properties. Prior literature has shown direct implications of the pore size and porosity of scaffolds on cell proliferation, differentiation, and ECM production [19,22]. Through experiments, we found that the size of macro-pores was determined by the digital model of 3D-printed scaffolds, while the size of micro-pores was determined by the annealing temperature of lyophilization. Hierarchical porous scaffolds offer multiple benefits, including facilitating cell ingrowth, promoting nutrient exchange, providing topological cues for cell attachment, and offering adsorption sites for bioactive molecules [22,23]. Three-dimensional printing also helped to achieve a uniform pore size of 400 μm, with optimized biofunction as previously reported [20,21]. Moreover, we investigated the influences of annealing temperatures on pore sizes of the scaffolds. Scaffolds were separately annealed at different temperatures (−20 °C, −80 °C and −178 °C), and the results showed that the inner connectivity of pores and the distribution of pore size were simultaneously optimized at −80 °C. Therefore, −80 °C was set as the cryo-storage temperature.

Mesenchymal stem cells (MSCs) are well-suited for regeneration due to their pluripotent capabilities, including self-renewal and differentiation. Among MSCs derived from various tissues, BMSCs demonstrate high proliferative ability and have been widely investigated in treatment of cartilage repair [24,25]. Implanting cell-free scaffolds with bioactive molecules can promote the migration and differentiation of endogenous mesenchymal stem cells, offering an alternative approach for in situ cartilage regeneration [7]. The key factors for successful regeneration include creating a conducive microenvironment and ensuring an adequate number of stem cells in the defect area. Appropriate incorporation of bioactive molecules is the key step of the functionalization of the hydrogel scaffolds. Multiple bioactive molecules have been applied in previous studies to recruit BMSCs onto the cartilage scaffolds, including chemokines, growth factors, etc. [21]. In this study, we enhanced the SF/MAHA scaffold by conjugating E7, a BMSCs-specific-affinity peptide. The peptide demonstrated a high affinity for MSC-like cells in the bone marrow, enabling selective capture and efficient entrapment [15]. Both microscopic observation and CCK-8 assay exhibited improved capacities of cell recruitment, and proliferation after E7-modification. In vivo experiments further visualized its superior ability of cartilage regeneration.

Regarding clinical applications, results of this study helped to confirm the efficacy of cell-free strategy in tissue-engineered treatment of cartilage defects. Compared with cell-loaded transplantation, cell-free scaffolds reduce potential risks of donor-site morbidity, and raise significantly less concerns over the isolation, expansion, preservation, and delivery of stem cells [26]. As a representative of cell-free paradigm, peptide-functionalized hydrogel scaffolds may arouse further attention and have great prospects in future clinical practice.

## 4. Materials and Methods

### 4.1. Preparation of SF and MAHA

Bombyx mori cocoon (15 g) were cut into pieces and boiled in 0.02 M Na_2_CO_3_ solution for 30 min to remove sericin. The extracted silk was dissolved in 9.3 M lithium bromide solution at 60 °C for 4 h and subsequently dialyzed using benzoylated dialysis tubing (MWCO: 2 kDa) in distilled water for 48 h. After two centrifugation steps at 9000 rpm and 4 °C for 20 min each, the product was dispensed and lyophilized for long-term storage [27].

The HA (Yuanye biotechnology, Shanghai, China) was modified with double bonds by reacting it with methacrylate anhydride (MA, Macklin, Shanghai, China). In summary, 1 g of HA was dissolved in 100 mL of water until fully dissolved. Then, 1 mL of MA was added, and the reaction was allowed to proceed for 12 h at a pH of 8–8.5. After stirring at 0 °C for 24 h, the MAHA was precipitated by centrifugating at 4000 rpm for 10 min in cold ethanol and subsequently dialyzed against water for 48 h. The purified MAHA was then lyophilized for storage [22].

### 4.2. Preparation of 3D-Printed SF/MAHA Scaffolds

MAHA was dissolved in deionized (DI) water at a concentration of 0.8% to create solution “A”. Similarly, Irgacure 2959 (BASF, Ludwigshafen, Germany) was dissolved in DI water at a concentration of 0.8% to create solution “B”. After dissolving SF in solution B, solutions A and B were mixed by centrifuging at 1000 rpm for 1 min to remove bubbles and impurities. The resulting mixture was then poured into the printing tube and crosslinked using a CL-1000 Ultraviolet crosslinking box (UVP, Upland, CA, USA) for 40 min. The printing process was carried out using a three-dimensional bioplotter (Envision Tec., Gladbeck, Germany). A 23 G dispensing needle with cylindrical tip was chosen. The printing pressure was set from 2.5 bar to 4.0 bar, and the printing speed was set between 6 mm/s and 15 mm/s. Three-dimensional printed hydrogels were annealed at different temperature (−20 °C, −80 °C, −176 °C) for 24 h and lyophilized. Dried scaffolds were immersed in ethanol for 2 h. For short-term storage, the scaffolds could be reserved in 75% ethanol solution. Prior to cell seeding, the scaffolds were washed with sterile PBS for 28 h to ensure the removal of any residual ethanol.

### 4.3. Functionalized Modification of the Scaffolds

Peptide E7 (EPLQLKM) and fluorescein 5-isothiocyanate (FITC) labelled E7 were commercially synthesized (Scilight-Peptide Ink., Beijing, China). To enable conjugation to the scaffolds, an additional cysteine residue was attached to the carboxyl terminus. Prior to conjugation, the scaffolds were initially immersed in a 10 wt% solution of 1,6-hexanediamine in isopropanol at 37 °C for 1 h. Subsequently, they were washed with PBS buffer three times. Next, the scaffolds were immersed in a Sulfo-SMCC solution (2 mg/mL in PBS buffer) at 37 °C for 1 h, followed by another three washes with PBS buffer. Once reacted with Sulfo-SMCC, the scaffolds were immersed in the peptide solution and incubated overnight at 4 °C. Afterwards, the scaffolds were thoroughly washed with PBS buffer and then lyophilized for storage [23].

### 4.4. Characterization of the Scaffolds

#### 4.4.1. Nuclear Magnetic Resonance (NMR)

The degree of MAHA was measured using a Bruker 600 MHz 1H NMR, following a previously reported method [24]. MAHA samples were dissolved in D_2_O at a concentration of 0.1%. To determine the degree of modification, the area ratio of proton peaks at 5.6 and 6.1 ppm (methacrylate protons) to the peak at 1.9 ppm (N-acetyl glucosamine of HA) was calculated.

#### 4.4.2. Fourier Transform Infrared Spectroscopy (FTIR)

Samples were crushed to powder and prepared using the KBr disk method. FTIR spectra were then acquired using an Affinity-1S FTIR spectrometer (SHIMADZU, Kyoto, Japan) with a resolution of 4 cm^−1^ and a scanning frequency of five times.

#### 4.4.3. Measurement of Peptide Conjugation Rate

The conjugation rate of E7 was measured following Huang’s protocol, utilizing FITC-labeled E7 for quantitative assessment. After lyophilization, the dry weight of the scaffold was recorded. The scaffolds were then immersed in 1 mL peptide solution with concentrations ranging from 0.05 mg/mL to 0.35 mg/mL. Following conjugation, the scaffolds underwent thorough washing and were subsequently digested using a 2 mL proteinase K solution (0.4 U/mL) for a duration 3 days. The fluorescence intensities of the digestion were measured at 490 nm (excitation) and 525 nm (emission) using a Varioskan Flash reader (Thermo Fisher Scientific Inc., Wilmington, DE, USA). The concentration of FITC-E7 in the digestion was calculated by referring to an FITC-E7 standard curve (*n* = 5).

#### 4.4.4. Morphology

The surface and internal cross-section of the scaffolds were examined using scanning electron microscopy (SEM). The scaffolds were freeze-dried and sputter-coated with gold via a Gatan Model 691PIPS (Gatan, Pleasanton, CA, USA). Images of the surface and internal morphologies were gathered using a FEI Quanta 200F SEM (FEI, Eindhoven, The Netherlands) at 15 KV accelerating voltage.

#### 4.4.5. Mechanical Characterization

Rheological measurements were performed using a MARS rheometer (HAAKE, Vreden, Germany) equipped with a cone rotor (20 mm diameter, 1° cone angle, 52 μm gap). An oscillatory time sweep test was conducted to determine the storage modulus and loss modulus at 10% strain and 1 Hz for a duration of 60 s. The printed samples had dimensions of 10 mm × 10 mm × 1 mm (*n* = 5).

#### 4.4.6. Enzymatic Degradation Profile

Proteinase K (Biofroxx, Einhausen, Germany) solution was used for in vitro enzymatic degradation. After recording the dry weight of the lyophilized scaffolds (*W*_0_), they were immersed into the enzyme solution (2 mL, 0.04 U/mL) at 37 °C. The solution was replaced with fresh enzyme solution every 2 days. At days 1, 2, 3, 4, 5, 7, and 10, the remaining scaffolds were taken out, washed with PBS buffer 5 times, lyophilized and weighed (*W*_d_). Mass residual was calculated as the ratio of *W*_d_ divided by *W*_0_ (*n* = 5).

### 4.5. Assessment of Biocompatibility of the Scaffolds

#### 4.5.1. Isolation and Identification of BMSCs

BMSCs were isolated following a previously reported protocol [15]. Briefly, bone marrow was extracted from the femur and tibia of New Zealand white rabbits. The extracted cells were then incubated in α-minimal essential medium (α-MEM) supplemented with 10% fetal bovine serum (FBS), 100 U/mL penicillin and 100 mg/mL streptomycin with 5% humidified CO_2_ at 37 °C. Non-adherent cells were eliminated by refreshing the culture medium after 3 days of incubation. Upon reaching confluence, typically after 4 to 5 days of culture, the adherent cells were considered as passage 0.

#### 4.5.2. BMSCs Seeding on the Scaffolds

The scaffolds underwent sterilized using cobalt-60 for 24 h and were additionally exposed to ultraviolet (UV) light for 1 h prior to being utilized in a laminar flow bench for cell seeding [25]. Each scaffold was seeded with 1 × 10^6^ BMSCs in 50 μL of medium (DMEM + 10%FBS). After 2 h incubation period for cell adhesion, fresh medium was added and subsequently replaced every 3 days.

#### 4.5.3. In Vitro Recruitment Capacity and Cell Viability

To assess the specific cell recruitment capacity of the E7 peptide, the effluent of cells was counted at 12 h and 24 h after implanting BMSCs on the scaffolds (*n* = 6). After 3 days of culture, the viability of BMSCs on different scaffolds were examined using confocal microscopy and Live/Dead staining (Invitrogen, Carlsbad, CA, USA). The cells were immersed in a 1 mL working solution containing 2 mM calcein-AM and 4 mM ethidium homodimer-1 reagents, followed by incubation at room temperature for 1 h. Visualization of live cells (green fluorescence) was achieved with an excitation wavelengths of 488 nm, while dead cells (red fluorescence) were visualized using an excitation wavelength of 568 nm.

Cell Counting Kit-8 (CCK-8, Sigma, St. Louis, MO, USA) was chosen to quantitatively measure the proliferation of BMSCs seeded on different scaffolds following the manufacturer’s protocol. The same amount of BMSCs were seeded on different scaffolds, including SF/MAHA scaffolds, SF/MAHA + E7 scaffold, and scaffolds consisted of MAHA with SF of different concentrations. Following coculturing for 1, 3, 5 and 7 days, 50 μL of CCK-8 solution was introduced to the medium and incubated for 4 h at 37 °C. Subsequently, the optical density (OD) value was measured at 450 nm using a plate reader.

#### 4.5.4. Morphology of BMSCs on the Scaffolds

The morphology of BMSCs on both scaffolds was observed using confocal microscopy (Leica, Nussloch, Germany) (Figure 4b)). To achieve this, the two BMSC-loaded scaffolds were washed with PBS three times for 5 min and then fixed with 4% paraformaldehyde for 30 min at room temperature. The cytoskeleton of the BMSCs was stained with Rhodamine-Phalloidin (160 nM; Cytoskeleton Inc., Denver, CO, USA) for 1 h 37 °C. After three additional washes, the nuclei were counterstained with Hoechst 33, 258 (2 μg/mL; Fanbo, Beijing, China) for 10 min.

#### 4.5.5. Biochemical Analysis of Glycosaminoglycan (GAG) and DNA Content

Chondrogenic differentiation was induced using the OriCell chondrogenesis differentiation kit (Cyagen Biosciences, Guangzhou, China), which includes 1X ITS (insulin-transferrin-selenium), dexamethasone, l-ascorbic acid, l-proline, sodium pyruvate and transforming growth factor β3. The cell/scaffold constructs were digested overnight in papainase (125 μg ml^−1^) at 60 °C. The GAG content was quantified using a 1,9-dimethylmethylene blue (DMMB) assay. Lysates (20 μL) were mixed with 200 μL of DMMB working solution and incubated at room temperature for 30 min. The absorbance at 535 nm was measured. Chondroitin sulfate (Sigma) served as the standard for GAG quantification. The DNA content was assessed using the Hoechst 33258 assay (Beyotime Biotechnology, Beijing, China). In summary, a 20 μL lysate was combined with 200 μL of Hoechst 33258 working solution and incubated at 37 °C for 1 hour. The absorbance was measured at 360 nm for excitation and 460 nm for emission. Calf thymus DNA (Sigma) served as the standard. The GAG or DNA content was expressed as micrograms per milligram of wet weight.

### 4.6. Animal Experiments

Animal experiments were conducted following the approval of the institutional biomedical ethics committee (LA2022554) and in accordance with the guidelines for the Care and Use of Laboratory Animals (National Academies Press, National Institutes of Health Publication No. 85-23, revised 1996).

#### 4.6.1. Surgical Operation

For in vivo experiments, 18 New Zealand White rabbits weighing 2.5–3.0 kg (four to six months) were used. The rabbits were anesthetized with 10 mL of ethyl carbamate (0.2 g/mL) through the ear vein. The right knee was prepared by shaving, sterilized with iodine solution, and draping in a sterile manner. An arthrotomy was performed through an anteromedial parapatellar incision, and the patella was everted. A 4mm diameter and 1mm depth cartilage defect was created in the trochlear groove of the distal femur using a corneal trephine [28].

The rabbits were randomly divided into three groups. In the microfracture (MF) group (*n* = 6), the osteochondral defects were left empty, and microfracture was performed into the medullary cavity, serving as control group. In the SF/MAHA group (*n* = 6), the cartilage defect with microfracture was implanted with SF/MAHA scaffolds. In the SF/MAHA + E7 group *(n* = 6), the corresponding defect was implanted with SF/MAHA + E7 scaffolds (Figure 5a). All scaffolds were positioned at the level of the adjacent cartilage surface, followed by repositioning of the knees. Subsequently, forced knee flexion and extension were performed to verify the precise placement of the scaffolds within the defect. For each group, 3 animals were selected for additional experiments at 6 and 12 weeks after the surgery, respectively.

#### 4.6.2. Magnetic Resonance Imaging (MRI)

Rabbits in each group underwent MRI analysis using a Siemens TIM Trio 3.0 T MRI scanner (Siemens, Erlangen, Germany) at 6 and 12 weeks after the surgery to detect neocartilage formation. Spin-echo sequences, as previously described, were employed for the MRI examinations [23].

#### 4.6.3. Histological Analysis

Distal portions of the rabbit femurs were fixed in 4% paraformaldehyde (pH = 7.4) for 48 h at 4 °C. The specimens were then decalcified using decalcifying solution (Zhongshan Goldenbridge Co., Ltd., Beijing, China) for approximately 5 days, until they could be easily punctured with needles. After the demineralization process was completed, the decalcified specimens were trimmed, embedded in paraffin, and sectioned into 5 μm slices. hematoxylin-eosin and toluidine blue staining were performed on the paraffin sections. Immunohistochemistry was also conducted using antibodies against collagen II (COL II) (Calbiochem, Boston, MA, USA).

### 4.7. Statistical Analysis

Statistical analyses were conducted using SPSS version 22.0. The data distribution is evaluated by Shapiro-Wilk test. The data are checked for equal variance before analysis. One-way analysis of variance (ANOVA) and post hoc tests were used for multiple comparisons The data were presented as mean ± standard deviation from a minimum of three independent experiments. A significance level of *p* < 0.05 was considered statistically significant. 

## 5. Conclusions

Herein, we synthesized a dual-crosslinked SF/MAHA scaffold using 3D printing technology, which was further functionalized with a BMSC-specific-affinity peptide E7 conjugated onto the scaffold. The scaffold showed a hierarchical porous morphology, excellent mechanical property and suitable biodegradation profile, providing a promising biomaterial for articular cartilage repair. In vitro, the SF/MAHA + E7 scaffold exhibited good biocompatibility and promoted the recruitment, proliferation and matrix production of BMSCs. In vivo, superior capacity of cartilage repair was displayed by the SF/MAHA + E7 scaffold as compared with MF and unconjugated SF/MAHA scaffold, especially at late stage. Macroscopic observation, histologic analysis and MRIs comprehensively suggested better integration between neocartilage formation and the uninjured area in the E7-functionalized scaffold. These findings demonstrated great potential of the SF/MAHA + E7 scaffold for cartilage regeneration in situ, which may inspire the development of other structurally and functionally optimized scaffolds and bio-implantation applications.

## Data Availability

The data that support the findings of this study are available from the corresponding author upon reasonable request.
